# Liberia—Its Fevers—Longevity of Its Inhabitants

**Published:** 1844-09

**Authors:** 


*Liberia—its Fevers—Longevity of its Inhabitants.*—At the semi-monthly meeting, May, 1st, 1844, of the Medical Department of the National Institute, Dr. Lindsly, to whom the subject had been previously referred, presented the following abstract of a communication from James L. Day, A.M., M.D., late physician to the colony of Liberia in western Africa, and United States agent for taking care of recaptured Africans—and, on motion, the abstract was ordered to be published.

To the question, what has been the effect of agriculture, the felling and clearing off the forests, the draining and cultivation of the soil, upon the climate, upon the health of the inhabitants, and upon the character of disease?

He states that it is only about twenty-two years since the colony of Liberia was first established—that during that period something has been effected (quite as much as under the circumstances could be expected) in the cutting down of the forests, draining the swamps, and in cultivating the soil—and that owing to these improvements and the better modes of treating disease, the acclimating fever, as it is called, which attacks almost all the emigrants, is much milder in its character than formerly, seldom confining the colored man more than a few days, and then leaving him in perfect health—and very frequently not proving dangerous even to the white, in whom it is generally very easily controlled by the administration of appropriate remedies.

The endemic, commonly called the African or coast fever, originates no doubt from the malaria generated from the extensive mangrove swamps almost everywhere present along the coast, combined with that generated from the vegetation everywhere luxuriant and decaying with great rapidity in this warm country, especially in the season of the periodical rains. This once assumed the character of a malignant bilious remittent, rarely taking the milder form of intermittent or tertian agues. Recently, from the causes before suggested, when remittent it is more mild, and oftener it appears in the intermittent form. In general it yields readily to remedial agents, and it is found the best practice one in which mild mercurial cathartics are used by day and sedatives at night, avoiding the danger of bringing on, by drastic purgatives, fatal hypercatharsis—and likewise avoiding entirely the use of the lancet, except possibly sometimes it may be employed in cases of sailors of full plethoric habit. But even in such cases, Dr. Day remarks, he would resort to it with fear and reluctance, At the first intermission, and in some cases the first considerable abatement, without waiting for a distinct intermission of the febrile symptoms, after the system has been brought under the action of calomel, he gives the sulph. of quinine, in doses of from five to twenty grains, according to the circumstances of the case. After having once prepared the system and commenced the use of the quinine, he remarks, he is rarely compelled by the return of the fever to stop it, except during the after part of the day, for the first or second day of its employment. It is continued in small doses for about three days, and then administered, dating from the interrupted paroxysm of the fever, on the sixth, thirteenth, and twentieth days thereafter, there being a constant tendency in such fevers at septemary periods.

What is the relative degree of health and longevity of the whites and blacks, the increase and mortality of each?

In answer to this question, Dr. Day remarks that he considers that the colored man, in emigrating to Africa from America, incurs much less risk to health and life than he would in going to Canada or the West Indies—that the cold of the former would produce more severe and more fatal diseases than are encountered in Africa, while the yellow fever of the West Indies, which is entirely unknown to Liberia, is much more dangerous than the mild acclimating fever to which the negro is liable upon landing in Africa. Upon the whole, he contends that the danger incurred by the colored emigrant is very slight indeed; and that even the white man is much less exposed than is generally imagined, many having lived in the colony four, five, six, seven and eight years in tolerable health, while many slave dealers have grown gray in the pursuit of wealth by their horrid traffic.—*Boston Med. and Surg. Jour.*, *June* 12.

